# Bone Marrow Aspiration Does Not Induce a Measurable Pain Response Compared to Sham Procedure

**DOI:** 10.3389/fvets.2018.00233

**Published:** 2018-10-01

**Authors:** Aileen L. Rowland, Cristobal Navas de Solis, Mauricio A. Lepiz, Kevin J. Cummings, Ashlee E. Watts

**Affiliations:** ^1^Department of Large Animal Clinical Sciences, Texas A&M University, College Station, TX, United States; ^2^Department of Small Animal Clinical Sciences, Texas A&M University, College Station, TX, United States; ^3^Department of Population Medicine and Diagnostic Sciences, Cornell University, Ithaca, NY, United States

**Keywords:** MSC, anesthesia, pain, heart rate variability, bone marrow aspiration, cortisol, horse

## Abstract

Bone marrow is commonly collected from horses for regenerative medicine applications. Little information is available regarding pain experienced by the horse during bone marrow aspiration. The objective of this study was to characterize horse reaction and pain response during bone marrow aspiration (BMA) compared to a sham (SHAM) procedure. We hypothesized there would be significantly greater horse reaction or pain response measured by salivary cortisol, heart rate variability, and depth and duration of sedation between BMA and SHAM. Twelve university owned horses underwent a BMA and sham procedure, 4 weeks apart in a randomized cross-over design, while sedated with 0.4 mg/kg xylazine hydrochloride. As measures of sedation depth, head height was recorded and sedation level was scored at specific procedural time points. Salivary cortisol was measured immediately before and 2 h after each procedure. Heart rate variability was assessed before, during, and after each procedure. There were no differences in head height, sedation score, or salivary cortisol between groups. No differences were noted between groups in heart rate variability before or during the procedure, but there was a significant decrease in low frequency/high frequency (LF/HF) ratio after the procedure in the BMA group. Over time, there was a significant reduction in LF/HF ratio during the procedure in both groups. Overall, BMA from the sternum did not result in a measurable pain response during, or in the 2 h following the procedure, in comparison to a sham procedure.

## Introduction

Bone marrow is collected from the equine sternum for therapeutic regenerative medicine applications. Although the anatomy of the equine sternum and the procedure for bone marrow aspiration (BMA) are well known, there is little information available regarding the pain response in the horse (1). In humans, bone marrow aspiration is known to be a painful procedure that is sometimes performed under general anesthesia to mitigate anxiety and pain (2–4). However, in our clinical experience there is little to no observable pain response from horses during BMA, and general anesthesia, or other analgesics such as NSAIDs or opiates are not required. We routinely use local anesthetic to eliminate sensation from the skin, subcutaneous tissue, and periosteum and mild sedation to decrease horse reaction to environmental stimulus, thereby minimizing horse movement and ensuring clinician safety.

We sought to evaluate horse reaction and pain response during BMA by evaluating sedation depth and duration during the procedure as measured by a sedation score and head height; heart rate variability (HRV) before, during, and after the procedure; and salivary cortisol prior to and after the procedure. HRV is a sensitive and validated measure of stress and pain in the horse (5, 6). Salivary cortisol is a commonly used objective measure of stress or pain with peak cortisol concentrations reached 120 min after stimulus (7–11).

To account for reaction to restraint, instrumentation, environmental stimulus, and sedation we compared BMA to a sham procedure in a randomized cross-over design where horses were instrumented and sedated but no other procedures were performed. We hypothesized that BMA would result in a significantly greater pain response measured by depth and duration of sedation, head height, sedation score, heart rate variability and salivary cortisol, between a BMA and sham procedure.

## Materials and methods

Twelve university-owned horses were used in the study. Horses were cared for according to the principles outlined in the NIH Guide for the Care and Use of Laboratory Animals and this study was approved by the institution's animal care and use committee (IACUC 2015-0038). Horses were held in stalls for approximately 1 h before and a minimum of 2 h after the BMA and sham procedure. Thereafter, horses were returned to group housing in paddocks and monitored daily for 1 week.

### Study design

The study design was a randomized crossover where half of the horses (*n* = 6) had a BMA performed first followed by a sham procedure (SHAM) 1 month later and vice versa. Two BMA and two SHAM procedures were performed each day, with horse assignment to BMA or SHAM procedure randomized prior to the start of the first procedure. The second procedure for each horse was performed in the same treatment room, on the same day of the week, and at the same time of day.

For each procedure, Holter monitors (Televet,® Heusenstamm, Germany) were placed and recording began at least 30 min prior to procedure start. After 30 min of recording with the Holter monitor, horses were walked into the treatment room, placed in stocks, and sedated with 0.4 mg/kg xylazine hydrochloride (Anased,® Akron, Inc., Lake Forest, Illinois, USA) intravenous through direct venipuncture: this was *t* = 0. Immediately after sedation, saliva was collected to measure salivary cortisol, and again 2 h after the end of the procedure. A handler remained present at the horse's head and the horse remained in the stocks for a minimum of 30 min or until the end of the procedure and was then returned to a box stall for 2 h. If additional restraint, sedation, or local analgesic was administered (at the discretion of the veterinarian performing the BMA or supervising the SHAM) it was recorded. Stocks were fitted with a centimeter (cm) scale for determining head height. Both procedures were video-recorded and recordings were used later to determine sedation scores and head height.

### Bone marrow aspiration

An area of the sternum approximately 10 cm long and 8 cm wide and centered on midline at the level of the elbow was clipped and aseptically prepared. Thirty mls of 2% lidocaine (Vetone,® distributed by MWI, Boise, Idaho, USA) was injected subcutaneously to the level of the periosteum. An 11-gauge × 4 inch BMA needle was used to collect 60 ml total of bone marrow from the sternum as previously described (1). A board certified surgeon with extensive experience performing bone marrow aspirations performed all procedures.

### Sham procedure

Horses were kept in the stocks for a total of 30 min. A handler was present at the horse's head as well as near the left elbow, mimicking the presence of people during the BMA procedure; however, no other aspects of the procedure were performed such as clipping of the hair or injection of lidocaine.

### Sedation score and head height

Sedation score and head height were evaluated by two independent observers using the video recordings of BMA and SHAM procedures. Time of intravenous xylazine administration was set as *t* = 0. The procedural steps for scoring during BMA were the initial observation (Initial) prior to xylazine administration, aseptic site preparation (Prep) which began immediately after xylazine administration, injection of local anesthesia (Block), BMA, and 5 and 10 min after completion of BMA. For the SHAM procedure, time points were matched to average BMA time points, Initial at *t* = −1 min, Prep at *t* = 5 min, Block at *t* = 10 min, BMA at *t* = 15, 5 min post at *t* = 20 and 10 min post at *t* = 25.

Head height was measured at the poll against a cm scale affixed to the stocks (Supplementary Figure 1). Sedation scores were assigned using a simple descriptive scale modified from Erber et al. for a total maximum score (most sedate) of 10 (12). Individual scores were assigned for horse attitude (nervous, 0; calm, 1; apathetic, 2; stuporous, 3), horse stance (stands well, 0; leans lightly, 1; leans heavily, 2; difficulty standing/ataxic, 3), horse head position (moving, 0; quiet, 1; hanging in halter, 2), horse eye position (normal, 0; partially closed, 1), and horse ear position (moving, 0; not moving, 1).

### Salivary cortisol

Saliva was collected using a commercially available salivary collection swab (Salivette,® Sarstedt, Germany) that was placed in the buccal space for 1 min as previously described (7, 13). Collection swabs were stored at room temperature for 2 h prior to being centrifuged at 1000 g for 2 min and the resulting saliva frozen at −80°C for 30–60 days prior to analysis. Salivary cortisol concentrations were determined in all samples at the same time according to manufacturer instructions of a commercially available ELISA (Salimetrics, Carlsbad, CA).

### Heart rate variability analysis

A Holter monitor was applied in a modified base-apex lead configuration 30 min prior to the horse entering the stocks and removed 2 h after the end of the procedure. Heart rate was recorded continuously and 10 min segments were analyzed beginning 20 min prior to entering the stocks (pre-procedure), during the procedure (procedure), and 2 h after the procedure (post-procedure). Commercially available equipment and software (Televet,® Heusenstamm, Germany) was used to record ECG information, and then to analyze heart rate variability of the recorded data (Kubios® HRV, Kuopio, Finland). Normal R-R intervals were analyzed with a 30% artifact correction in lead II using Televet® software as previously described (6). Abnormal rhythms, including 2nd degree AV block, were excluded from analysis. In Kubios®, the artifact filter was set to low, 0.35 s, which removed any abnormal beats that were missed during manual correction in Televet®. Analysis and frequency trends were set as previously reported (6). Briefly, lambda was set at 500 ms (ms), removing very low frequency trends, Fast Fourier Transform spectrum was set to a 50% window overlap at 512 s, LF was set to 0.01–0.07 Hz, and HF was set to 0.07–0.6 Hz. Mean heart rate, standard deviation of the NN (R-R) intervals (SDNN), proportion of the number of pairs of successive NN (R-R) intervals that differ by more than 50 ms divided by the total number of NN (R-R) intervals (PNN50), the root mean square of successive heartbeat interval differences (RMSSD), low frequency (LF), high frequency (HF), and low to high frequency power ration (LF/HF) were all evaluated. Additionally, prior to correction, mean heart rate was counted for all time points with the inclusion of abnormal rhythms.

### Statistical analyses

Data was imported into commercially available statistical software program (GraphPad Prism,® Ja Jolla, California, USA) for analysis. Mean head height and sedation score was derived from the scores of both observers. Head height, sedation scores, cortisol concentrations, and heart rate variability parameters were check for normality and analyzed over time using repeated measure analysis of variance (RM-ANOVA) and follow up analysis of differences between groups using a Sidak's multiple comparisons. A paired *T*-test was used to compare differences in cortisol concentrations between groups and between time points. A Cohen's Kappa analysis was performed to evaluate inter-observer agreement of assigned sedation scores. For all analyses, *p* ≤ 0.05 were considered significant.

## Results

We used 10 American Quarter Horse mares, 1 Quarter Horse type mare, and 1 American Quarter Horse gelding. The median age was 12 years old (range 6–20 years old). The median total procedure time from injection of sedation to the completion of BMA was 28 min (range, 22–44 min). One horse was excluded from head height and sedation score analysis because video recording of the entire BMA was not obtained due to a recording error. One horse required additional sedation (0.2 mg/kg xylazine) and application of a nose twitch prior to injection of local anesthetic, and one horse required 10 ml of additional local anesthetic due to continued skin sensation that was noted by the clinician because of skin twitching and abdominal tensing during penetration of the skin by the needle.

### Sedation score

**Figure 1:**
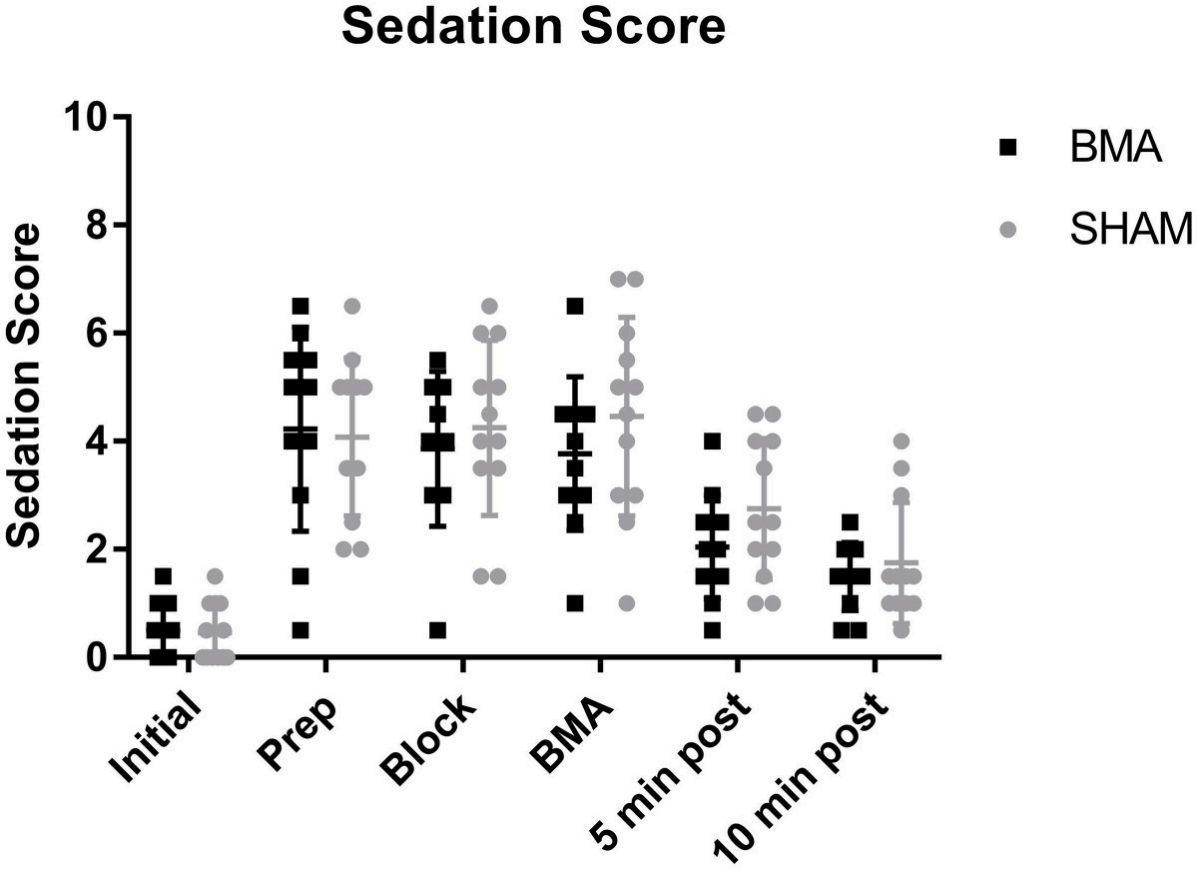
Sedation scores during BMA (black boxes) and SHAM (gray circles) procedures. All horses became sedate after xylazine administration with significantly higher sedation scores during Prep, Block, and BMA compared to Initial (*p* = 0.001). There were no significant differences between groups at any time point.

All horses became sedate with significantly higher sedation scores after xylazine administration (Prep through BMA time points) and significantly lower sedation scores (less sedate) after the procedure (5 and 10 min post-procedure), that remained significantly higher than Initial values (*p* = 0.001). There was no difference in sedation score between BMA or SHAM procedures at any time point (Figure 1). Cohen's Kappa of inter-observer variability was fair with a value of 0.31, however, it should be noted that one observer scored consistently higher than the other.

### Head height

**Figure 2:**
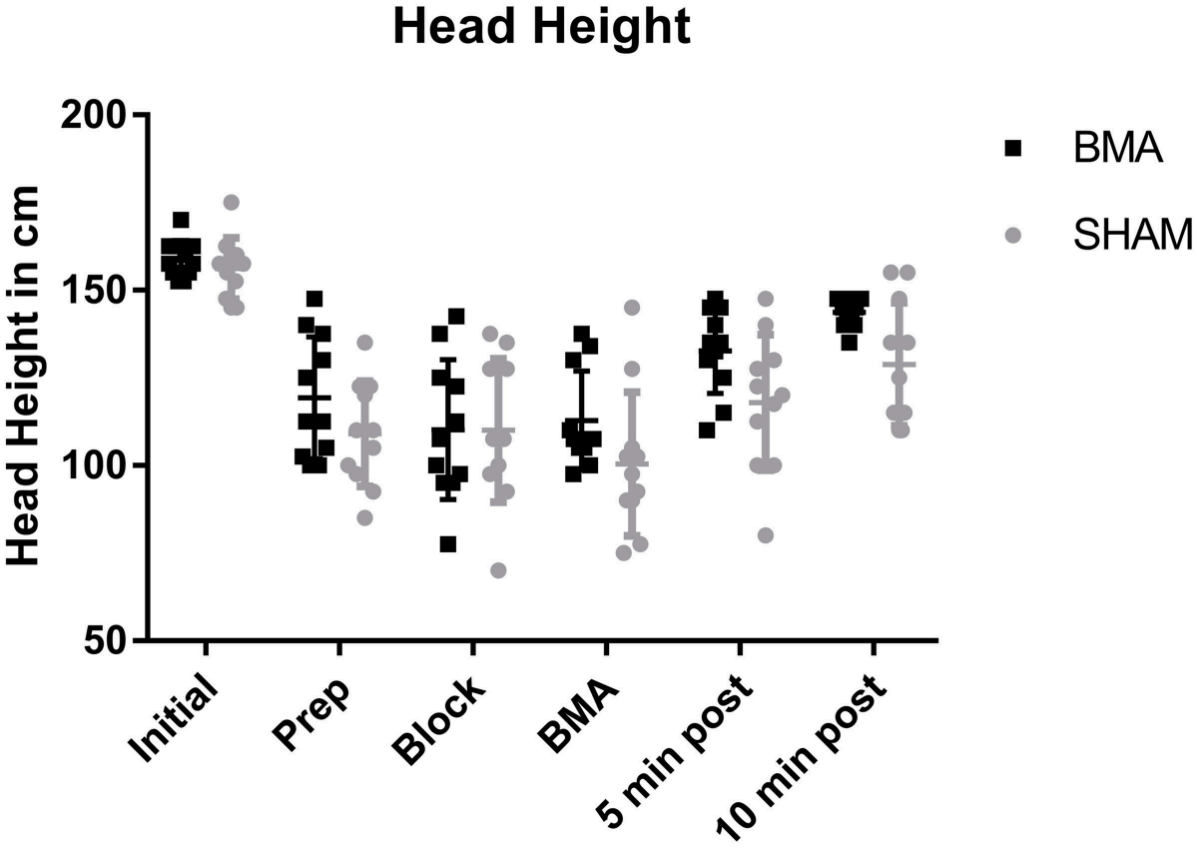
Head height during BMA (black boxes) and SHAM (gray circles) procedures. There was a significantly lower head height during Prep, Block, and BMA (*p* < 0.001) which is reflective of the degree of sedation after xylazine administration. There were no significant differences between groups at any time point.

All horses became sedate with significantly lower head heights after xylazine administration (Prep through BMA time points) and significantly higher head heights (less sedate) after the procedure (10 min post), that remained significantly lower than Initial values (*p* < 0.001). There was no significant difference in head height between BMA or SHAM procedures at any time point (Figure 2).

### Salivary cortisol

**Figure 3:**
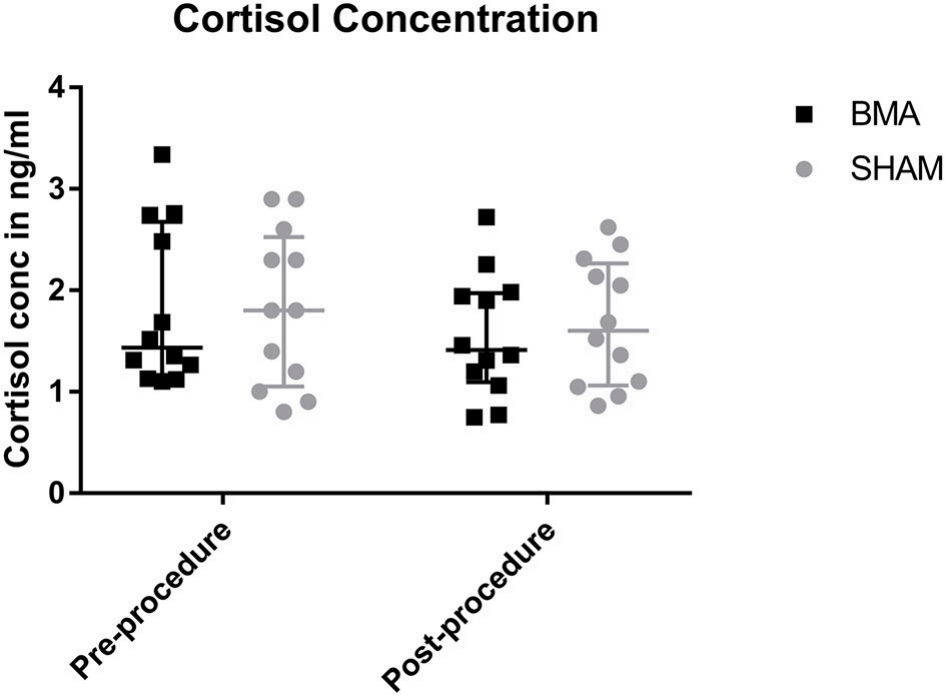
Salivary cortisol concentrations during BMA (black boxes) and SHAM (gray circles) pre-procedure and post-procedure. There were no differences between groups at either time point.

There was no significant difference in salivary cortisol concentrations between groups at either time point (pre-procedure and post-procedure) or between time points. Prior to the procedure median salivary cortisol concentration was 1.1 ng/ml in the BMA group, and 1.8 ng/ml in the SHAM group (BMA range, 1.1–3.3 ng/ml; SHAM range, 0.8–2.9 ng/ml) (Figure 3). After the procedure median salivary cortisol concentration was 1.4 ng/ml in the BMA group, and 1.7 ng/ml in the SHAM group (BMA range, 0.7–2.7 ng/ml; SHAM range, 0.9–2.6 ng.ml) (Figure 3).

### Heart rate variability analysis

**Figure 4:**
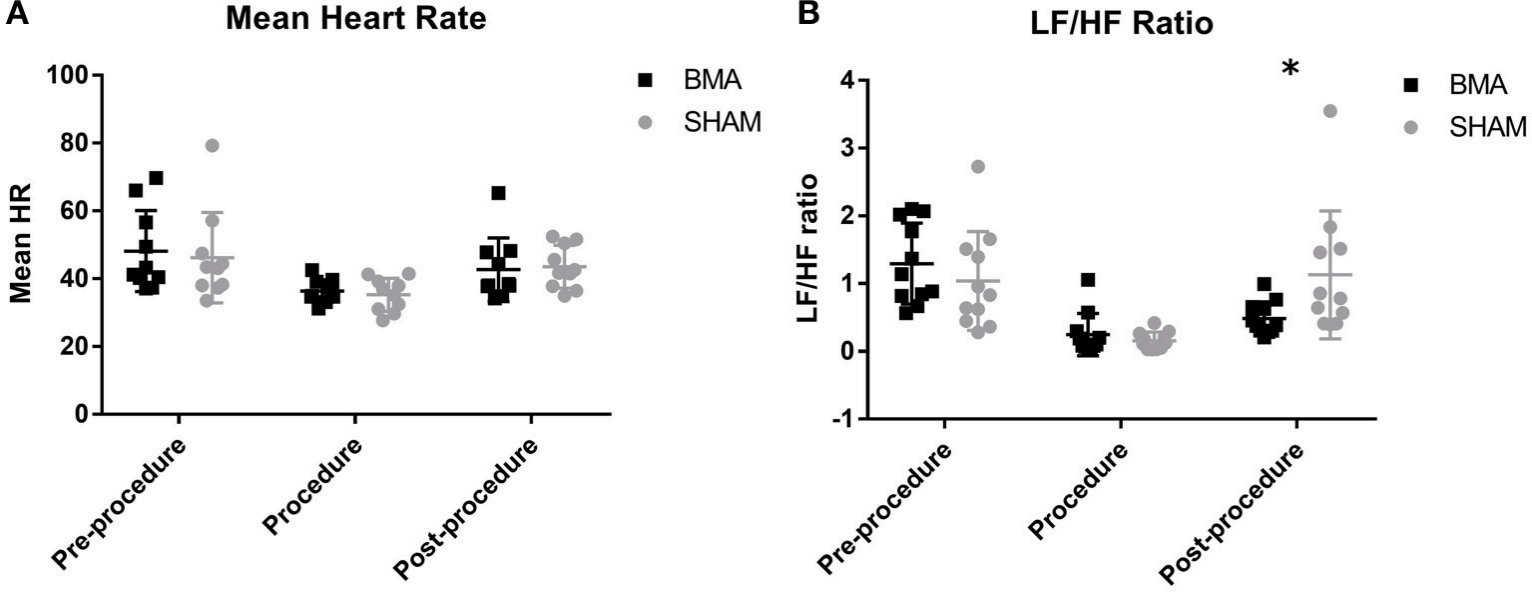
Heart rate variability between BMA (black boxes) and SHAM (gray circles) procedures. **(A)** Mean heart rate pre-procedure, during the procedure and post-procedure. There were no differences between groups at any time point. There was a significant decrease in HR during the procedure compared to pre-procedure in both groups (BMA, *p* = 0.02; SHAM, *p* = 0.04). **(B)** Low frequency/high frequency (LF/HF) ratio pre-procedure, during the procedure and post-procedure. Between groups there were no differences during pre-procedure and procedure. Post-procedure, a significant reduction was noted in the BMA group compared to the SHAM group (p = 0.03). When evaluated over time, there was a significant reduction in LF/HF ratio noted in both groups during the procedure compared to pre-procedure values (BMA, 0.001; SHAM, *p* = 0.005).

Complete data was not available for one horse during BMA procedure and for a different horse during SHAM procedure because of recording error so HRV data from these procedures for these horses was excluded. When comparing mean heart rate there was no significant difference between groups at any time point (Figure 4A). Over time, there was a significant decrease in mean heart rate between pre-procedure and procedure in both groups (BMA, *p* = 0.02; SHAM, *p* = 0.04) and mean heart rate post-procedure remained significantly lower than pre-procedure but was not different to procedure. When heart rates were manually counted, there were also no differences between groups during the procedure (BMA median 36 bpm, range 31 - 44; and SHAM median 32 bpm, range 28–41) or between procedure and post-procedure. For the frequency domain measure there was no significant difference between the groups at any time point except at the post-procedure time point where there was a significant decrease in LF/HF ratio (*p* = 0.03) in the BMA group (Figure 4B). Over time, there was a significant decrease in LF/HF ratio for both groups between pre-procedure and procedure time points (BMA, *p* = 0.001; SHAM, *p* = 0.005). There were no significant differences in LF or HF between groups or over time. There were no significant differences between groups or over time for the time domain variables of SDNN, PNN50, and RMSSD.

## Discussion

The purpose of our study was to evaluate horse reaction and pain response during BMA. To account for differences due to handling, instrumentation, environmental stimulus and sedation, we compared results during BMA to a sham procedure when horses were placed in stocks and sedated but no additional procedures were performed. We found no difference between groups at any time point in the objective measures of pain we evaluated: head height as a measure of sedation level, sedation score, or salivary cortisol. We also evaluated HRV, as a measure of sympathetic and parasympathetic tone and an indirect measure of pain and stress. There were no differences in HRV between the groups that would indicate increased pain response in the BMA group. We conclude there is no difference in reaction or pain response in mildly sedated horses undergoing BMA compared to SHAM procedure.

Heart rate variability is a very sensitive and specific measure of pain that has been used to evaluate treatment protocols in cases of acute laminitis, in evaluating pain response to hot branding and microchip implantation, and as a prognostic indicator in horses with colic (6, 11, 12). When evaluating HRV there are two main types of measurements: time domain variables and frequency domain variables (14). Time domain variables measure the differences in R-R intervals thus measuring beat to beat differences, while frequency domain variables measure frequency specific oscillations using a Fourier transformation algorithm which can allow it to be more sensitive to minute changes (14).

When comparing HRV between groups, we found no significant differences in time domain variables at any time point and no significant differences in frequency domain variables before and during the procedure. However, there was a significant decrease in the frequency domain LF/HF ratio, a measure of the balance between sympathetic and parasympathetic tone, in the BMA group after the procedure (15). Known to increase in painful horses with increased sympathetic tone, the LF/HF would have increased had the procedure induced significant or prolonged pain (6). Decreased LF/HF ratio can result from an increase in parasympathetic tone due to relaxation or administration of a parasympathomimetic medication or from a relative decrease in sympathetic tone (16). The difference in LF/HF ratio could be because horses in the BMA group had an increase in parasympathetic tone post-procedure due to lidocaine administration, a sodium channel blocker known to have parasympathomimetic effects when administered at 1.4 mg/kg (17, 18). Horses in the BMA group received ~1.2 mg/kg subcutaneously, while horses in the SHAM group did not receive lidocaine. Subcutaneous injection of lidocaine in humans has been shown to cause a significant decrease in LF/HF ratio similar to our results (18). In any case, the reduction in LF/HF does not support a painful response to the procedure.

When HRV was evaluated over time there were no differences in the time domain variables we evaluated: RMSSD, pNN50, or SDNN. Had there been long lasting pain or discomfort after either procedure the time domain measurements would have changed over time compared to baseline measurements (6). However, there was a significant decrease LF/HF ratio, during the procedure in both groups. This is because sedatives, including xylazine, are known to decrease the LF/HF ratio due to increased parasympathetic and decreased sympathetic tone (19).

While there is an abundance of literature evaluating serum cortisol levels in horses, salivary cortisol may be a better measure of pain response as it measures the unbound metabolically active portion, as opposed to serum cortisol, which measures both bound and unbound fraction (7). The salivary cortisol concentrations in our horses were not different due to treatment or over time and cortisol concentrations in both groups (1.4 and 1.7 ng/ml after BMA and SHAM procedures) were below the known resting salivary cortisol concentrations of 2 ng/ml (7, 10, 13). This indicates there was minimal stress or pain response in either group.

A limitation of any study of pain in animals, but especially horses, is that pain is difficult to objectively measure. Several systems exist, but many rely on physiologic parameters or emotional responses, which are altered when horses are sedated with alpha-2 agonists (20–23). We did not feel it was appropriate to perform BMA without sedation because of clinician safety while collecting sternal aspirates between the forelimbs of horses. This is because of unpredictable horse movement due to environmental stimulus. Since depth and duration of alpha-2 agonist sedation are related to the level of pain experienced by the horse, we used sedation depth and duration as indirect measures of pain (24). We expected that if there was significant pain experienced by the horses there would be significant differences in head height and sedation score with a shorter duration of sedation and lesser degree of sedation. Although we found differences in depth of sedation over time (scores and head height), we found no differences in depth or duration of sedation between groups. Related to the effects of pain on depth and duration of sedation, xylazine is known to have analgesics effects and may have masked mild pain in our horses (25).

There are potential limitations to HRV analysis. In order to analyze time domain and frequency variables accurately, abnormal R-R intervals (e.g., AV block) are removed. This can lead to falsely elevated mean heart rates in sedated horses because blocked segments are not used to calculate rate. To account for this we also performed manual counts of heart rate during the procedure and post-procedure. With this inclusion of irregular R-R intervals there were still no differences between groups during the procedure or between the procedure and post-procedure time points. Furthermore, the ability of HRV analysis to detect differences is influenced by the editing procedures during data analysis and the stringent parameters we used for artifact correction may have limited our ability to see differences (6). This may explain why there were no differences in LF and HF whereas the LF/HF ratio was different. Regardless, had we used less stringent parameters and detected changes in the LF and HF ratio we would not have changed our conclusion.

A final limitation of this report is that the sham procedure did not include clipping, aseptic preparation, or administration of local anesthetic. This precluded the individuals scoring the procedures from being blinded to the procedure, however, there was no difference noted between groups. Had we performed these additional aspects of the procedure during the sham we would have been even less likely to find a difference.

Bone marrow aspiration is increasingly commonplace in equine clinical patients for bone marrow-derived regenerative therapies. In people, BMA is understood to be a painful procedure despite use of local anesthetics and sedation. One might expect the pain response to be similar in horses; however, a strong correlation of pre-procedural anxiety level and reported pain during BMA in people have been found (26–28). Horses would not be expected to have the same response because of specific pre-procedural anxiety to BMA. Using salivary cortisol, heart rate variability, and depth and duration of sedation we determined that sternal BMA, with local anesthetic and mild sedation, was not different in pain response or reaction compared to SHAM during or for 2 h after the procedure in the horse. Therefore, analgesics such as NSAIDs or opiates should not be necessary during or after the procedure. This disparity in pain response between humans and horses might be useful for clinicians to accurately inform owners and trainers prior to performing BMA.

## Author contributions

ALR study design, data acquisition and analysis, and manuscript preparation; CN study design, data analysis, and manuscript preparation; ML analysis and interpretation of data, manuscript preparation. KJC analysis and interpretation of data, manuscript preparation; AEW study design, data acquisition, analysis and interpretation of data, manuscript preparation.

### Conflict of interest statement

The authors declare that the research was conducted in the absence of any commercial or financial relationships that could be construed as a potential conflict of interest.

## Supplementary material

Supplementary Figure 1 Representative images of a horse in the stocks next to the head height scale **(A)** prior to sedation, at **(B)** peak sedation, and at the **(C)** end of the sham procedure.
