# Supplementary figures and images for: Bone Marrow Aspiration Does Not Induce a Measurable Pain Response Compared to Sham Procedure
Source: Front Vet Sci. 2018 Oct 1;5:233. doi: 10.3389/fvets.2018.00233 (PMC6174850; doi:10.3389/fvets.2018.00233)

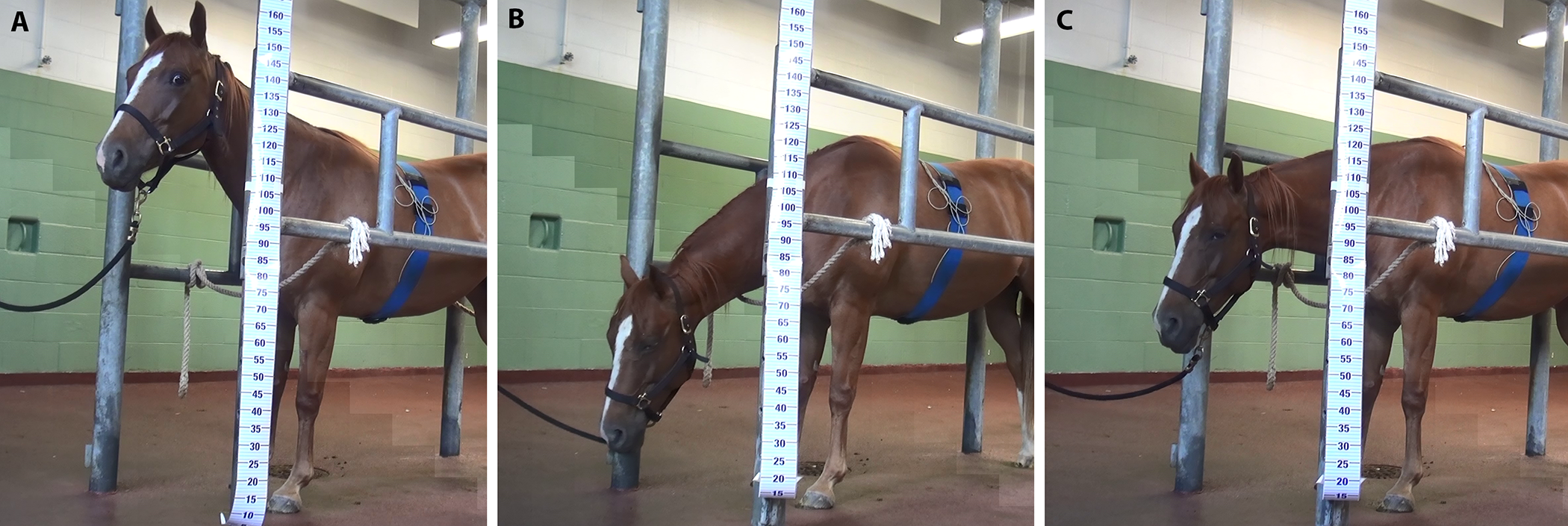

Supplement: Supplementary Figure 1 — Representative images of a horse in the stocks next to the head height scale (A) prior to sedation, at (B) peak sedation, and at the (C) end of the sham procedure. [file Image_1.TIF]
